# In People who Identify as Gender Minority People the Social Cure Model and in People who Identify as LGBTQ* People the Intragroup Status and Health Model might Explain the Link between Identity Centrality and Body Appreciation

**DOI:** 10.1192/j.eurpsy.2024.1158

**Published:** 2024-08-27

**Authors:** N. Komlenac, K. Stockburger, J. Birke, M. Hochleitner

**Affiliations:** Gender Medicine and Diversity Unit, Medical University of Innsbruck, Innsbruck, Austria

## Abstract

**Introduction:**

Sexual and gender minority (SGM) people are often found to have lower levels of body appreciation than do cis-heteronormative people.

**Objectives:**

The current study utilizes the social cure model and the intragroup status and health model to investigate whether identification with a SGM social group and identity centrality (i.e., the degree to which a specific social identity is important to an individual) is linked to experiences of hostile behaviors because of a person’s looks or body and consequently, to body appreciation.

**Methods:**

A cross-sectional online-questionnaire study was conducted with 1,680 German-speaking participants (49.2% cisgender women, 37.7% cisgender men, 9.0% non-binary, 4.1% transgender; *M*_age_ = 32.7, *SD* = 12.5). The Multidimensional and Multicomponent Measure of Social Identification, the Body Appreciation Scale-2, the Perceived Stigmatization Questionnaire and the Sociocultural Attitudes Towards Appearance Questionnaire-4, revised were used. A manifest path model was calculated.

**Results:**

People who identified as gender minority (GM) people and LGBTQ* people reported lower levels of body appreciation. Sexual minority (SM) individuals who identified with a social group other than LGBTQ* people reported levels of body appreciation similar to those of individuals who identified as women. Individuals who identified as GM people experienced fewer instances of hostile behaviors because of their looks or body the higher their level of identity centrality was. On the other hand, individuals who identified as LGBTQ* people more frequently encountered hostile behaviors because of their looks or body when their identity centrality was strong. Frequent experiences of hostile behaviors because of a person’s looks or body was linked to poor body appreciation in all social groups.

**Conclusions:**

Identity centrality might help alleviate experiences of discrimination, especially in people who identify as GM people, as the social cure model suggests. In line with the intragroup status and health model, individuals who strongly identify as LGBTQ* people might be more visible as SM people and experience more discrimination than do SM people who identify with another social group.

**Disclosure of Interest:**

None Declared

